# Reports from the Case Book of Thos. S. Powell, M. D., Sparta, Georgia

**Published:** 1856-05

**Authors:** 


### ARTICLE II.

*Reports from the Case Book of* Thos. S. Powell, M. D., Sparta, Georgia.

*Case* 1*st*. July 29th, 1847. I was called to see Mary, a colored woman of robust constitution, aged 35 years; found her suffering intensely with a constant deep seated pain in her left knee, which was very much swollen. Mrs. P., her mistress, informed me that about three weeks previous to my seeing her, she complained of a pain in her knee, and said it seemed to be in the bone, and that she, Mrs. P., supposed it to be a simple sprain; applied vinegar and clay from the back of the chimney, for several nights, which gave her temporary relief, but as it did not relieve her entirely, and her services were much needed in the field, she sent for Dr. ———, a celebrated Thompsonian, who resided a short distance from the place. He came, and pronounced it a case of white swelling, and treated her up to the day before I saw her. His treatment I did not learn, except that he vomited her severely, several times, with lobelia. With this statement, I examined the case minutely, and pronounced it a case of synovial inflammation. I prescribed nothing internally as the symptoms did not warrant it. As I was in the dilemma that country physicians are often placed in, fourteen miles from my office, and without the means of applying the remedies recommended by authors, I was somewhat puzzled to know how to proceed, but I remembered the old maxim, "Necessity is the mother of invention," and set to work and did the best that my judgment, saddlebags, and Mrs. P. could afford. In the first place, I found that I had 2ʒ of carb. ammonia, which I dissolved in one quart of apple vinegar, furnished by Mrs. P. Secondly, I ordered a strong decoction of mullen and pine top, and as soon as it was ready, I bathed the knee with it, as hot as she could bear it. I then enveloped the whole joint well with bats of wool, saturated with the vinegar and ammonia, warmed, and fixed it up nicely with a common roller. I ordered this to be done twice a day, morning and night. Gave some general directions, and left to return in a few days.

Aug. 4. Mary is decidedly better, very little pain, swelling nearly reduced, and able to move the joint without assistance. I prepared another bottle of vinegar and ammonia, and ordered it to be applied in the same way. Dismissed the case.

In 12 or 15 days, Mr. P. came to town and reported her well, and in the field at work.

*Case* 2. Joe Clark, a lad ten or twelve years of age, in the spring of 1854, was taken while at school, with a pain in his right knee. That evening, it was with difficulty that he walked home. When he retired at night, his mother applied some domestic poultice. Next day he was much worse, the pain deep seated and constant; the joint somewhat swollen. I saw him on the sixth day. He was in bed, unable to get up or move the knee joint. It was very painful and very much swollen. Considering it a case of synovial inflammation, I ordered the affected part to be bathed with warm water, and closely enveloped with fullers earth, clear of gravel, worked in a sufficient quantity of the ammonia and vinegar mixture, prepared as in case 1st. I recommended the application to be made morning and evening; gave some general directions, and left, to return on the fourth day; but on the third evening, heard that Joe was able to be up, and was walking about the house, and having other patients to visit, I thought it unnecessary to visit him, as I had promised. In ten or twelve days Joe was at school, as well as usual. His mother informed me that he commenced to improve from the first application.

*Remarks.*

Sometime previous to my treating the second case, I read in some medical journal, a case reported by Professor Horner, as having been cured by the application of fullers earth and chamber lye, suggested by an old lady who visited his patient, and at the same time, or subsequently, another case cured by the application of guano in the form of a poultice. The object of Professor Horner, if I mistake not, in publishing these cases, was simply to offer to the profession the guano as a substitute for the urine and clay, as it is evident that it was the ammonia that effected the cure. My object in reporting the above cases so briefly, is to offer the ammonia and vinegar as a substitute for both, as the guano has not reached many parts of the country, and as I am certain that the extreme modesty that characterizes medical men generally, will prevent the urine application. As an instance of the truthfulness of the foregoing assertion, I know a worthy and eminent physician, not a thousand miles off, who was applied to by a reputable and clever lady, for a prescription for scurvey—he wishing to know what had been done—asked her if she had used any remedies. Yes, said she; "I have been using chamber lye." The unexpected reply from the source whence it came, so took the medical gentleman aback, that he retired, after some pleasant conversation on some other subject, foreign to the scurvey, and the remedy used—wishing for the good of the race that there was no such disease as scurvey; as long as there *was*, and he couldn't *help* it, if the remedy she mentioned must be used, that some one would give it a more unexceptionable title.

